# Physiologically-based pharmacokinetic modeling for optimal dosage prediction of olaparib when co-administered with CYP3A4 modulators and in patients with hepatic/renal impairment

**DOI:** 10.1038/s41598-023-43258-9

**Published:** 2023-09-25

**Authors:** Dongmei Gao, Guopeng Wang, Honghai Wu, Jiawei Ren

**Affiliations:** 1 Department of Medical Oncology, Bethune International Peace Hospital, Shijiazhuang, 050082 China; 2 Zhongcai Health (Beijing) Biological Technology Development Co., Ltd., Beijing, 101500 China; 3 Department of Clinical Pharmacy, Bethune International Peace Hospital, Shijiazhuang, 050082 China; 4 North China Electric Power University, No.2, Beinong Road, Huilongguan, Changping District, Beijing, 102206 China

**Keywords:** Clinical pharmacology, Pharmacokinetics, Pharmacology

## Abstract

This study aimed to develop a physiologically-based pharmacokinetic (PBPK) model to predict the maximum plasma concentration (C_max_) and trough concentration (C_trough_) at steady-state of olaparib (OLA) in Caucasian, Japanese and Chinese. Furthermore, the PBPK model was combined with mean and 95% confidence interval to predict optimal dosing regimens of OLA when co-administered with CYP3A4 modulators and administered to patients with hepatic/renal impairment. The dosing regimens were determined based on safety and efficacy PK threshold C_max_ (< 12,500 ng/mL) and C_trough_ (772–2500 ng/mL). The population PBPK model for OLA was successfully developed and validated, demonstrating good consistency with clinically observed data. The ratios of predicted to observed values for C_max_ and C_trough_ fell within the range of 0.5 to 2.0. When OLA was co-administered with a strong or moderate CYP3A4 inhibitor, the recommended dosing regimens should be reduced to 100 mg BID and 150 mg BID, respectively. Additionally, the PBPK model also suggested that OLA could be not recommended with a strong or moderate CYP3A4 inducer. For patients with moderate hepatic and renal impairment, the dosing regimens of OLA were recommended to be reduced to 200 mg BID and 150 mg BID, respectively. In cases of severe hepatic and renal impairment, the PBPK model suggested a dosing regimen of 100 mg BID for OLA. Overall, this present PBPK model can determine the optimal dosing regimens for various clinical scenarios involving OLA.

## Introduction

Olaparib (OLA) is a first-in-class poly ADP-ribose polymerase (PAPR) inhibitor and was approved in 2014 by FDA^1^. It is clinically indicated for the treatment of patients with ovarian or breast cancer^1,2^. In clinic, a 400 mg capsule formulation twice daily (BID) (16 × 50 mg large capsules) was first approved for treatment^3^. Afterwards, a tablet formulation of 300 mg BID (4 × 150 mg tablet) was developed to enhance patient compliance by reducing the number and size of units required to achieve a therapeutic dose^3^. The approved tablet dosage strengths for patients are 100 mg and 150 mg^2^.

OLA is partially hepatically cleared and metabolized primarily by CYP3A4^1^. Additionally, Approximately 44% of OLA is cleared by the kidney and excreted through urine^4^. The in vitro study showed that OLA was efficiently transported by human ABCG2 and ABCB1 (P-gp)^5^. Moreover, in the in vitro assessments, OLA showed the inhibition of multiple hepatic and renal uptake transporters, suggesting the strongest inhibition against transporter MATE1 with an IC_50_ of 5.5 μM^6^. Furthermore, in a study, OLA significantly induced CYP3A4 enzyme expression^7^.

The in vitro study revealed that a concentration of 1 μM OLA (equivalent to 435 ng/mL) is required for effective inhibition of various cancer cell lines, including those with BRCA mutant, low BRCA expression, and no-BRCA expression^4,8^. Notably, for cell lines with low BRCA and no-BRCA mutant expression, higher concentrations of OLA were necessary to achieve comparable clinical efficacy^4^. This is likely because, in cell lines expressing higher levels of BRCA mutations, the DNA damage repairment heavily relies on base excision repair, in which PARP plays a crucial role. In contrast, in cell lines exhibiting lower levels of BRCA mutations, DNA repair can still occur through the homologous recombination repair mechanism. As a result, higher concentrations of OLA are necessary to achieve desired efficacy. Additionally, in a BRCA2 breast cancer mouse model, tumor reduction was only observed at doses that sustained exposure above the IC_50_ for more than 13 h^4^. Furthermore, these findings from the in vitro study have been also further supported by clinical trials conducted in humans^4^. In the clinic, no statistically significant difference was observed in the overall response rate (ORR) and progression-free survival (PFS) between the 400 mg BID and 200 mg BID doses^4,9^. As a result, the 200 mg BID was identified as the lowest efficacy dose. The mean trough concentration (C_trough_) of OLA at 200 mg is 960 ng/mL^10^, 500 ng/mL^11^, and 855 ng/mL^12^ in the three clinical research studies, respectively. As a result, the pharmacokinetic (PK) threshold for optimum clinical efficacy was identified as a mean C_trough_ of ≥ 772 ng/mL. In a retrospective study^13^, there was a strong correlation between OLA exposure and early adverse events in patients with BRCA1/2 mutations. As a result, a C_trough_ of ≤ 2500 ng/mL was identified as a clinical safety PK threshold. The analyses of C_max_-anemia relationship in patients have suggested that the frequency of anemia in patients can reach approximately 55% when the C_max_ is above 12,500 ng/mL^4^. Overall, for optimal clinical efficacy and a safe profile, C_trough_ should be limited within 772–2500 ng/mL, and C_max_ is below 12,500 ng/mL.

The human AUC, C_trough_ and C_max_ of OLA can be influenced by multiple factors, including concomitant use with CYP3A4 perpetrators (drug–drug interactions, DDIs), and use in patients with insufficient hepatic/renal function. Previous studies have shown that co-administration of OLA with itraconazole (a strong CYP3A4 inhibitor) increased its AUC by 42%^3^, while co-administration with rifampicin (a strong CYP3A4 inducer) decreased its AUC by 72%^3^. Additionally, the clinical study also showed that patients with renal impairment had a 1.75-fold increase in AUC and a 1.39-fold increase in C_max_ of OLA compared to patients with normal renal function^14^.

When administering OLA to patients in various clinical scenarios, such as concurrent use with other CYP3A4 modulators or in patients with hepatic/renal impairment, it may be necessary to make dosing adjustments. When determining the optimum dosing regimens for OLA, it is important to consider the C_trough_ and C_max_ threshold as a key factor. To aid in this process, a PBPK model was developed in patients to (1) predict the C_trough_ and C_max_ of OLA in Caucasian, Japanese and Chinese patients, respectively; (2) predict C_trough_ and C_max_ alterations of OLA when combined use with CYP3A4 modulators, and use in Caucasian patients with insufficient hepatic/renal function; and (3) recommend an optimal dosing regimen in multiple clinical situations according to the C_trough_ and C_max_ threshold.

## Materials and methods

### Virtual population demographic characteristics

The demographic characteristics utilized in each simulation were derived from the corresponding clinical study. PK-Sim incorporates information such as age range, body weight, height, and proportion of female individuals from the virtual population data. In cases where the clinical studies do not provide specific demographic characteristics, similar studies are referenced to obtain relevant data for the simulations. In situations where certain data are unavailable, PK-Sim employs default values or values from similar studies as substitutes. To ensure an adequate sample size, if the number of subjects in the clinical studies is less than 10, 10 virtual subjects are created for the simulations. The demographic characteristics of the virtual population used in the simulations ae summarized in Table 1.

**Table 1:** Demographic data of race in virtual population.

Races	Clinical studies	Number of subjects	Age range	Proportion of female (%)	Population
Caucasian	Fong et al.^10^	17	19–82	67	Patients
	Dean et al.^11^	10	22–71	100	
			33–60	75	
	Mateo et al.^22^	17	35–75	100	
	Plummer et al.^23^	27	29–71	87	
Japanese	Yamamoto et al.^12^	10	54–67	33	Patients
			39–69	66	
	Yonemori et al.^20^	10	37–55	57	
			44–64	100	Patients
Japanese	Yuan et al.^21^	20	50–65	65	

### Software

The PBPK model was constructed using the PK-Sim® (Version 10.0, Bayer Technology Services, Leverkusen, Germany); PK profiles of OLA from published papers were digitized using Digit (Version 1.0.4, Simulations Plus, USA), and the figures were drawn using Origin 2019 (version 9.6.5.169, OriginLab, USA).

### PBPK model development

The three parameters (K_p_ scale, partition coefficients, and cellular permeabilities) are crucial in predicting drug distribution in body. In PK-Sim, five different methods are employed to determine tissue distribution: Rodgers and Rowland, PK-Sim standard, Schmitt, Poulin, Theil, and Berezhkovskiy. Cellular permeability, on the other hand, is calculated using two methods: PK-Sim standard and Charge dependent Schmitt. To enhance the alignment of predicted OLA concentration–time profiles with observed PK profiles, the distribution calculation for OLA in the PBPK model was optimized using the parameter identification module in PK-Sim. The method selected for tissue distribution calculation was Rodgers and Rowland, while the PK-Sim standard method was chosen for cellular permeability calculation. Furthermore, to further improve the agreement between predicted and observed PK of OLA, the K_p_ scale was optimized to 1.5. OLA has mean plasma protein binding of 89% observed at multiple concentrations^4^, f_up_ was hence set at 0.11. There were no reports indicating that kidney transporters or tubules are involved in the influx or efflux of OLA. Therefore, the fraction of GFR was set to 1.0. The efflux transport of OLA was described by intrinsic transport velocity (CL_int_). The P-gp CL_int,u_ was estimated as 0.63 μL/min/million cells^−1^ from the P_eff_ (effective permeability) data in transfected MDCKII/h ABCB1^5^. Similarly, ABCG2 CL_int,u_ was calculated to be 0.27 μL/min/million cells^−1^ in transfected MDCKII/h ABCG2 using the same method^5^. The renal clearance (CL_R_) was calculated using the glomerular filtration rate (GFR) × f_up_ method in PK-Sim®. The PBPK model of OLA contains one metabolizing enzyme (CYP3A4) and two efflux transporters (ABCB1 and ABCG2). Because the PK-Sim® expression database did not include the reference concentrations of the two transporters, the reference concentration for ABCB1and ABCG2 were calculated to be 0.68 and 0.13 μM/L liver tissue, respectively, using the formula (transporter abundance × expressed organ weight)/liver volume)^15^.

OLA is clinically available in two formulations, namely capsule and tablet, and there are two notable differences observed between the in vitro dissolution and in vivo PK. Compared to tablets, OLA capsules exhibit a slower dissolution rate and lower bioavailability^16^. The Weibull time parameter is a modeling parameter that can be utilized to characterize the speed of drug dissolution. Therefore, Weibull times for capsule and tablet were set at 60 and 30 min, receptively. Additionally, because the clinical bioavailability study of tow dosage forms revealed that the AUC of 300 mg BID tablet was similar to that of 400 mg BID capsule^16^, The CYP3A4 CL_int,u_ values, which are associated with AUC, were assigned as 0.058 and 0.044 μL/min/pmol for capsule and tablet, receptively. Similarly, HLM CL_int,u_ (human liver microsome) values for capsule and tablet were assigned as 0.22 and 0.17 μL/min/mg protein, receptively^16^.

**Figure 1:**
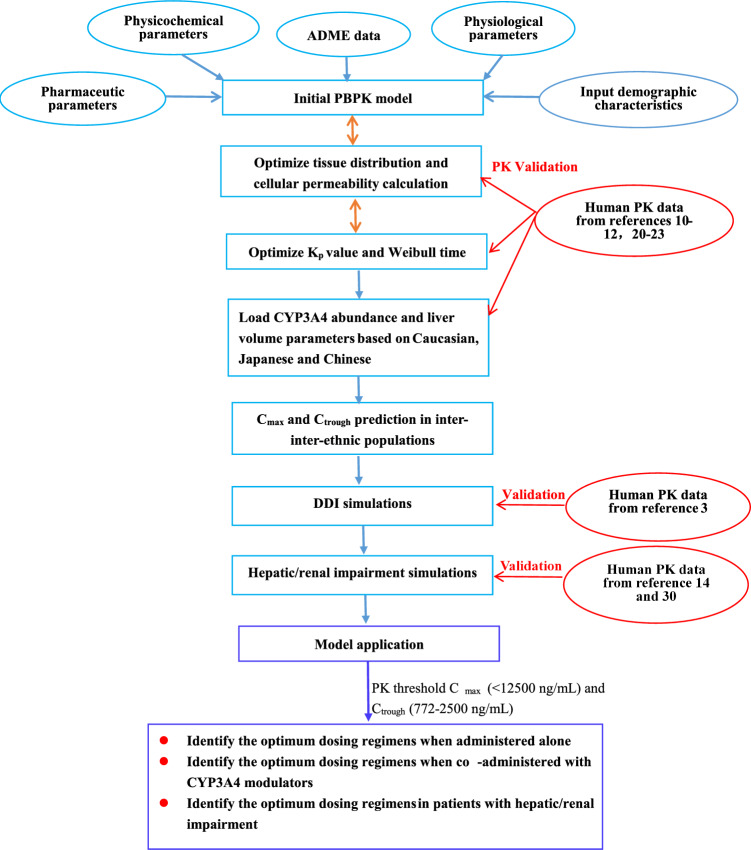
The generic workflow of the PBPK model for OLA. The population PBPK model was constructed using modeling parameters that encompassed the absorption, metabolism, and distribution processes associated with the CYP3A4 metabolizing enzyme, as well as the P-gp and ABCG2 transporters. The model was validated using multiple PK profiles in Caucasian, Japanese, and Chinese patients^10–12,20,21^. The model was then further verified using ratios between predicted and observed C_max_ and C_trough_^10–12,20–23^. DDIs and hepatic/renal impairment simulations were also validated using PK profiles in patients when co-administered with itraconazole and rifampicin^3^, using PK profiles in patients with hepatic/renal impairment^14,30^. Finally, the PBPK model was used to determine the optimal dosing regimens in various clinical scenarios.

When developing PBPK population models, the most commonly considered known inter-ethnic physiological differences include variations in height and weight distribution, metabolizing enzyme abundances, and liver volume^17^. In this PBPK model, the default liver volume was used at 2.38, 216 and 1.91 L for Caucasian, Japanese and Chinese, respectively. On the basis of literature data^18^, the CYP3A4 abundance was set at 137, 112 and 120 pmol/mg protein for Caucasian, Japanese, and Chinese, respectively. Table 2 summarizes the final parameters of the model, which were derived from various sources^1,4,5,7,16,18,19^. Figure 1 illustrates the generic workflow of the PBPK model.

**Table 2:** Summary of parameters used in the PBPK model.

Property (Units)		Values used in the model	Literature values and source	Descriptions
MW(g·mol^−1^)		434.46	Chemspider	Molecular weight
pKa (Acid)		12.07	12.07^19^	Acid dissociation constant
LogP		1.49	1.49^19^	Lipophilicity
Solubility (mg·mL^−1^)		0.12	0.12 (Water)^19^	Solubility in water
P_eff_ (× 10^–4^ cm s^−1^)		36.2	36.2^4^	Human effective permeability
f_up_		0.11	0.18^4^	Fraction of free drug in plasma
Rbp		0.80	0.80^1^	Blood-to-plasma concentration ratio
CYP3A4 CL_int,u_(μL/min/pmol)		0.058^a^, 0.044^b^	0.058^4,16^	Intrinsic clearance for CYP3A4 and HLM
Additional HLM CL_int,u_ (μL/min/mg)		0.22^a^, 0.17^b^	0.22^4,16^	
CYP3A4 abundance (pmol/mg protein)		137^c^,112^d^,120^e^	137^a^,112^b^,120^c^^18^	Content of CYP3A4 protein in liver microsomes
Liver volume (L)		2.38^c^, 2.16^d^, 1.91^e^	default	Liver volume
P-gp CL_int,u_ (μL/min/million cells^−1^)		0.63 (Estimated)	P_app_ (10^−6^ cm/s): 37.3 for P-gp and 27.4 for ABCG2^5^	Intrinsic transport velocity for P-gp and ABCG2
ABCG2 CL_int,u_ (μL/min/million cells^−1^)		0.27(Estimated)		
CL_R_(L/h)		GFR*f_up_	Default	Renal clearance
GFR fraction		1.0	Default	Fraction of filtered drug in the urine
K_p_ scale		1.5	Optimized	Organ-to-plasma partition coefficient
Partition coefficients		Rodgers and Rowland	Optimized	Calculation method from cell to plasma coefficients
Cellular permeabilities		PK-Sim Standard		Permeability calculation method across cell
Weibull time (min)		60^a^, 30^b^	Optimized	Dissolution time of 50% drug
Weibull shape		0.62	Default	Shape parameter of Weibull function
Concentration (μM/L liver tissue)	CYP3A4	4.32	Default	Reference concentration for metabolizing enzyme and transporters
	ABCB1	0.68	Calculated	
	ABCG2	0.13		
K_i_ CYP3A4 (μM)		72.2	14.3^7^	Reversible inhibition constant at CYP3A4
K_inact_ CYP3A4 (min^−1^)		0.068	0.072^7^	The maximum rate of inactivation against CYP3A4
EC_50_ CYP3A4 (μM)		18.0	18.0^7^	Half-maximal induction
E_max_ CYP3A4		57.6	57.6^7^	Maximum in vitro induction effect

^a^ Capsule, ^b^Tablet, ^c^ Caucasian, ^d^Japanese, ^e^ Chinese.

### PBPK model validation

The clinically observed PK data of OLA in Caucasian^10,11^, Japanese^12,20^, and Chinese^21^ patients were used to validate the predictive performance of the PBPK model by comparing the coincidence of predicted and observed PK profiles. Next, this model was also verified by comparing the ratios between the observed and predicted C_max_ and C_trough_^10–12,20–23^. Generally, the common acceptable criterion is between 0.5 and 2.0. It was assumed that steady state would be reached if OLA was taken BID for 14 consecutive days.

### Sensitivity analysis

The sensitivity analysis were performed to evaluate the impact of selected model parameters on the C_max_ and C_trough_, respectively. Patients were given standard dose regimens of 300 mg BID for 14 consecutive days. For the sensitivity analysis, both optimized and modeling parameters that were likely to have a significant influence on the C_max_ and C_trough_ were selected. The selected parameters were (1) LogP, (2) f_up_, (3) Rbp, (4) CYP3A4CL_int,u_ (5) P-gp CL_int,u_ and ABCG2 CL_int,u_, (6) K_i_ CYP3A4, (7) EC_50_ and E_max_ for CYP3A4 (8) expression (CYP3A4, P-gp, ABCG2), and (9) liver volume.

To evaluate the impact of the selected parameters on C_max_ and C_trough_, each parameter was altered by ± 20%^24^. The sensitivity coefficient (SC) is estimated as follows^24^:

1 $${\text{SC}} = \Delta {\text{Y}}/{\text{Y}} \div \Delta {\text{P}}/{\text{P}}$$

where ∆Y represents the alteration of predicted C_max_ or C_trough_; Y represents the start value of predicted C_max_ or C_trough_; ∆P is the change of modeling parameters; P represents start value of parameters. If a certain SC absolute value is greater than 1.0, it indicates that a 20% change in the evaluated parameter will result in a 20% change in predicted C_max_ or C_trough_. It signifies the model parameter has a significant impact on C_max_ or C_trough_.

### DDI simulations

The final modeling parameters of five modulators are given in supplementary Table S1. Table 3 summarizes the inhibition and induction parameters of five modulators^7,25–27^. In validating the DDI simulations, based on the literature’s data^3^, the dosage regimens of OLA were designed as 300 mg OD in patients for consecutive 14 days. The dosage regimens of itraconazole and rifampicin were set at 200 mg OD and 600 mg OD, respectively, for 10 days (from day 5 to day 14). The virtual demographic data for DDI simulations involving itraconazole and rifampicin were sourced from a published paper^3^. The DDI simulations were first validated by comparing the differences between the predicted and observed PK profiles when concurrently administered with itraconazole and rifampicin^3^. Subsequently, the PBPK model for OLA was integrated with the PBPK models of CYP3A4 inhibitors (ketoconazole, itraconazole, and fluconazole) and inducers (rifampicin and efavirenz), respectively, to predict the C_max_ and C_trough_ of OLA when co-administered with five CYP3A4 modulators.

**Table 3:** The inhibition and induction parameters of modulators.

Modulators	K_i_ (μM)	EC_max_	EC_50_ (μM)
Itraconazole^25^	0.0013 (CYP3A4)	–	–
Hydroxy-itraconazole^25^^a^	0.0023 (CYP3A4)	–	–
Ketoconazole^26^	0.015 (CYP3A4)	–	–
Fluconazole^27^	16.6 (CYP3A4)	–	–
Rifampicin^7^	–	121 (CYP3A4)	0.92
Efavirenz^b^	–	5.2 (CYP3A4)	0.07

^a^metabolite of itraconazole; ^b^built in the PK-Sim®.

### Simulations in patients with hepatic/renal impairment

The physiological parameters and characteristics for patients with inadequate hepatic or renal function were obtained from published papers and are presented in supplementary Table S2,S3. The virtual population demographic characteristics are presented in supplementary Table S4,S5. In hepatic impairment simulations, the default liver volume is 2.38 L in normal population in the PK-Sim®. Based on literature ratio data^28^, liver volumes were calculated to be 2.12, 1.69, and 1.45 L in patients with wild, moderate and severe hepatic impairment, respectively. According to the paper^29^, the fractional CYP3A4 CL_int,u_ value in patients with severe impairment was 0.4 of that in normal humans. The CYP3A4 abundance ratio of moderate to severe impairment was used to calculate the fractional CYP3A4 CL_int,u_ value (0.72) in moderate impairment patients. The actually determined albumin levels in normal, wild, and moderately impaired patients were obtained from the paper^30^. Albumin levels in severe impairment patients calculated using the ratio of severe to normal (0.6)^28^. The Plasma Protein Scale Factor (PPSF) in PK-Sim was utilized to account for variations in plasma albumin protein levels and unbound OLA fraction. PPSF is estimated^31^:

2 $$PPSF = \frac{1}{{f_{up} + \left( {1 - f_{up} } \right) \times {\text{Albumin}}_{f} }}$$

where Albuminf is the fractional value of plasma albumin in patients with hepatic/renal impairment with respect to normal individuals.

In renal impairment simulations, renal blood flow (RBF) is estimated by the following formula^32^:

3 $$\ln \left( {{\text{RBF}}} \right) = - 3 \times 10^{ - 5} \times GFR^{2} + 0.0170 \times GFR + 4.09$$

Kidney volume (KV) is estimated by the following formula^32^:

4 $$\ln \left( {{\text{KV}}} \right) = - 6.3 \times 10^{ - 5} \times GFR^{2} + 0.0149 \times GFR + 4.13$$

Based on the literature’s data^14,30^, The dosage regimens of OLA were set at 300 mg OD for patients with wild, moderate, and severe hepatic/renal impairment in the simulations. The simulations in patients with hepatic/renal impairment were validated by comparing the predicted and observed PK data^14,30^. Subsequently, the PBPK model was employed to forecast the C_max_ and C_trough_ values in patients with mild, moderate, and severe hepatic or renal impairment, respectively following multiple doses of OLA given over a period of 14 consecutive days.

### Human and animal rights

No human participants or cells were involved in this study, and the data were derived from publicly available sources.

## Results

### Validation of the PBPK model

**Figure 2:**
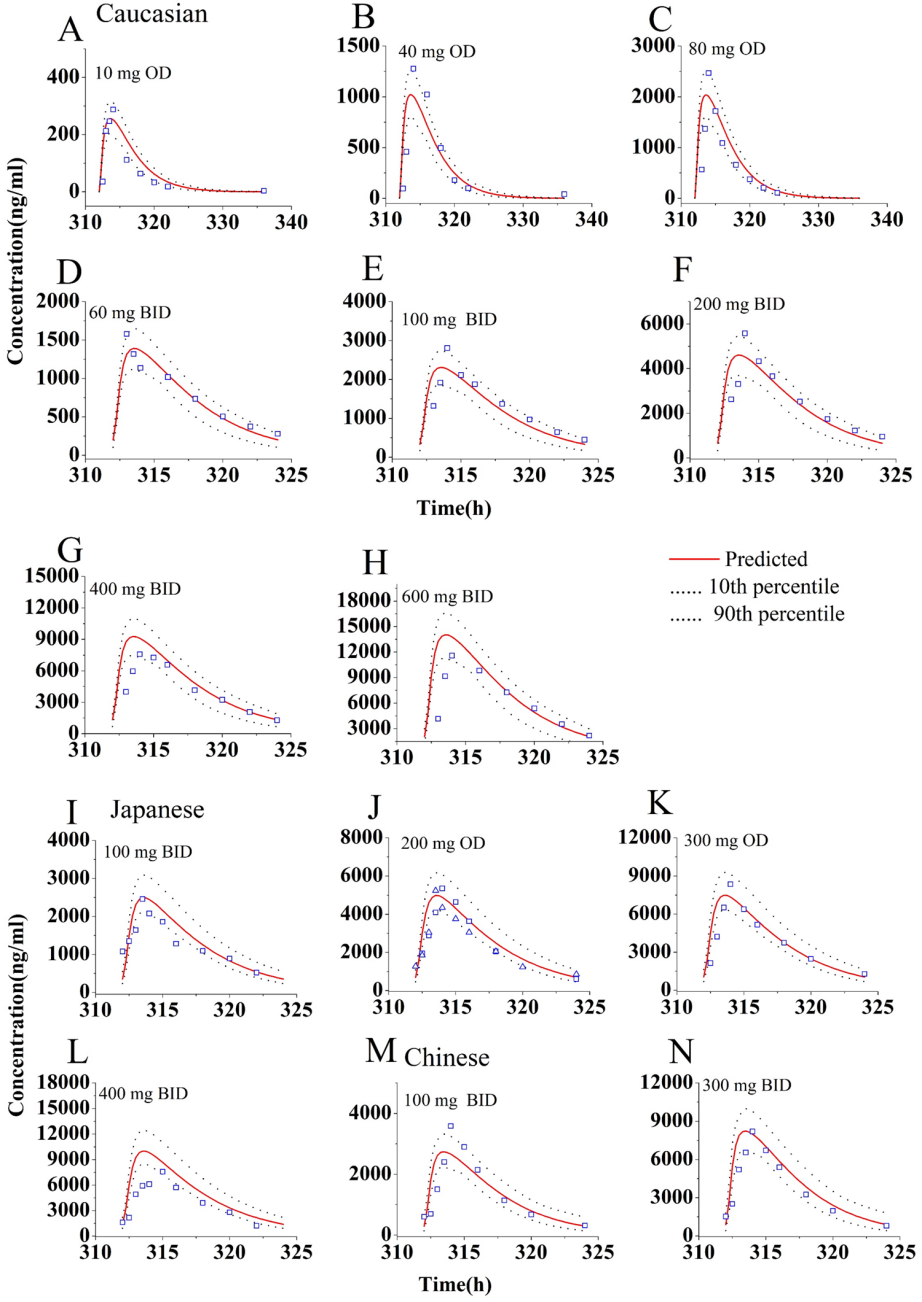
Simulations of the pharmacokinetics of OLA after administration of repeated doses. Blue squares and up-triangles are the clinically observed data.

Figure 2 shows the predicted and observed plasma concentration–time profiles in Caucasian (Fig. 2A–H), Japanese (Fig. 2I–L) and Chinese (Fig. 2M–N). The simulations showed that the developed population PBPK model may match the observed PK profiles^10–12,21,22^. As seen in Table 4, all the ratios of predicted and observed geometric mean C_max_ and C_trough_ were within 0.5–2.0, and most of predicted/observed ratios were in the range of 0.7–1.3. This suggests that this population PBPK model can predict accurately the C_max_ and C_trough_ of OLA at steady state in different population ancestry. Moreover, the ratios of prediction to observation were found to be between 0.69 and 1.69 in the Caucasian population, 0.72–1.70 in the Japanese population, and 0.79–1.37 in the Chinese population. These results indicate that there are no significant differences in the ratios among the three inter-ethnic groups.

**Table 4:** Comparisons of the geometric mean C_max_ and C_trough_ between predicted and observed data in different population ancestry.

Clinical study	Ancestry	Dose (mg BID) /dosage form	No. of subjects	Age range/mean (year)	Proportion of female	C_max_ (ng/mL, range/CV %^a^)		C_trough_ (ng/mL, range/CV %)		Prediction/observation ratio	
						Prediction	Observation	Prediction	Observation	C_max_ ratio	C_trough_ ratio
Fong et al.^10^	Caucasian	200/Cap^b^	17	19–82	0.67	4618 (3686–6854)	5620 (2830–17,100)	658 (276–999)	960 (210–2950)	0.82	0.69
		400/Cap	6			9283 (7418–13,806)	7650 (5280–10,500)	1340 (558–2029)	1290 (660–3890)	1.21	1.04
		600/Cap	5			14,034 (11,225–20,905)	11,500 (6490–17,600)	2072 (861–3126)	2180 (390–5950)	1.22	0.95
Dean et al.^11^		200/Cap	4	22–71	1.0	4747 (3530–6015)	4700 (35.6%)	698 (134–2055)	500 (191.2%)	1.01	1.40
		400/Cap	4	33–60	0.75	9750 (7468–12,080)	9100 (27.2%)	1544 (357–3721)	1600 (46.1%)	1.07	0.97
Mateo et al.^22^		400/ Cap	10	50.9	0.91	9659 (7244–12,075)	5710 (2380–10,900)	1489 (319–3872)	1860 (530–6670)	1.69	0.80
		400/ Cap	17	52.5	1.0	9428 (7487–11,458)	6360 (3880–13,300)	1398 (362–2992)	1040 (230–8490)	1.48	1.34
		200/Tab^c^	13	59.1	1.0	6605 (5159–8302)	6880 (4010–10,400)	892 (148–2813)	1000 (280–3100)	0.96	0.89
		300/Tab	17	55.8	1.0	9944 (7762–1250)	9370 (2280–14,700)	1358 (225–4254)	1840 (340–3830)	1.06	0.74
		400/Tab	10	54.2	1.0	13,320 (10,387–16,747)	12,000 (8450–16,900)	1845 (308–5735)	2010 (760–3610)	1.11	0.92
Plummer et al.^23^		300/Tab	27	29–71	0.87	10,062 (8091–12,379)	9500 (4800–19,700)	1444 (296–3697)	2000 (600–11,400)	1.06	0.72
Yamamoto et al.^12^	Japanese	200/Cap	3	54–67	0.33	4971 (4228–6177)	4800 (31.6%)	696 (460–1084)	855 (NR^d^)	1.04	0.81
		400/Cap	6	39–69	0.66	10,017 (8481–24,350)	5900 (19.7)	1413 (934–2202)	1220 (NR)	1.70	1.16
Yonemori et al.^20^		200/Tab	3	37–55	0.57	6480 (4453–9037)	7670 (46.9%)	675 (376–1357)	610 (157%)	0.84	1.11
		300/Tab	6	44–64	1.0	9749 (6693–13,604)	8430 (35.1%)	1025 (567–2065)	1290 (157.6%)	1.16	0.79
Yuan et al^21^	Chinese	300/Tab	20	50–65	0.65	11,303 (9482–16,019)	8270 (35%)	1019 (459–2838)	800 (118%)	1.37	1.27

^a^CV %, percentage coefficient of variation; ^b^Cap, capsule; ^c^Tab, Tablet; ^d^NR, not reported.

### Sensitivity analysis

**Figure 3:**
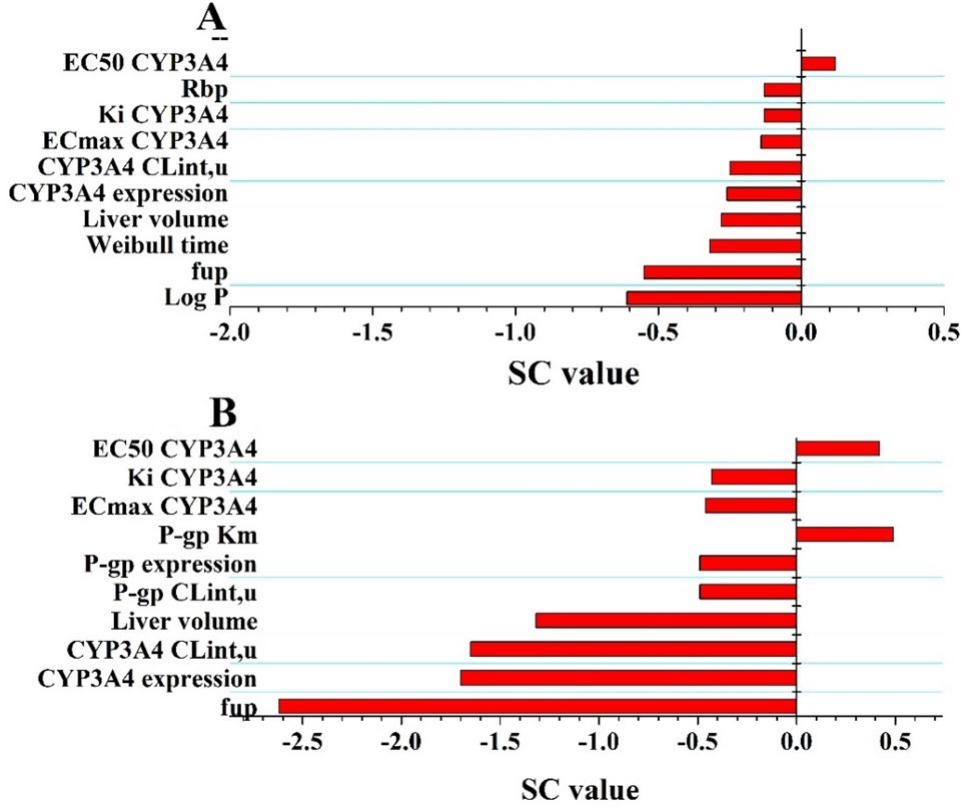
Sensitivity analysis of the PBPK model for C_max_ (**A**) and C_trough_ (**B**).

As shown in Fig. 3, the ten sensitive parameters to the OLA C_max_ (Fig. 3A) and C_trough_ (Fig. 3B) are represented. The most sensitive parameter was Log P (SC:-0.61) for C_max_ at steady-state. The sensitive parameters for C_trough_ were f_up_ (SC:-2.62), CYP3A4 expression (SC:-1.70), CYP3A4CL_int,t_ (SC:-1.65) and liver volume (SC:-1.32) at steady-state. Also of note was that four SC values for C_trough_ were more than 1.0. Overall, sensitivity analysis revealed the majority of modeling parameters had a slight impact on the C_max_ and C_trough_ of OLA.

### DDI simulations

**Figure 4:**
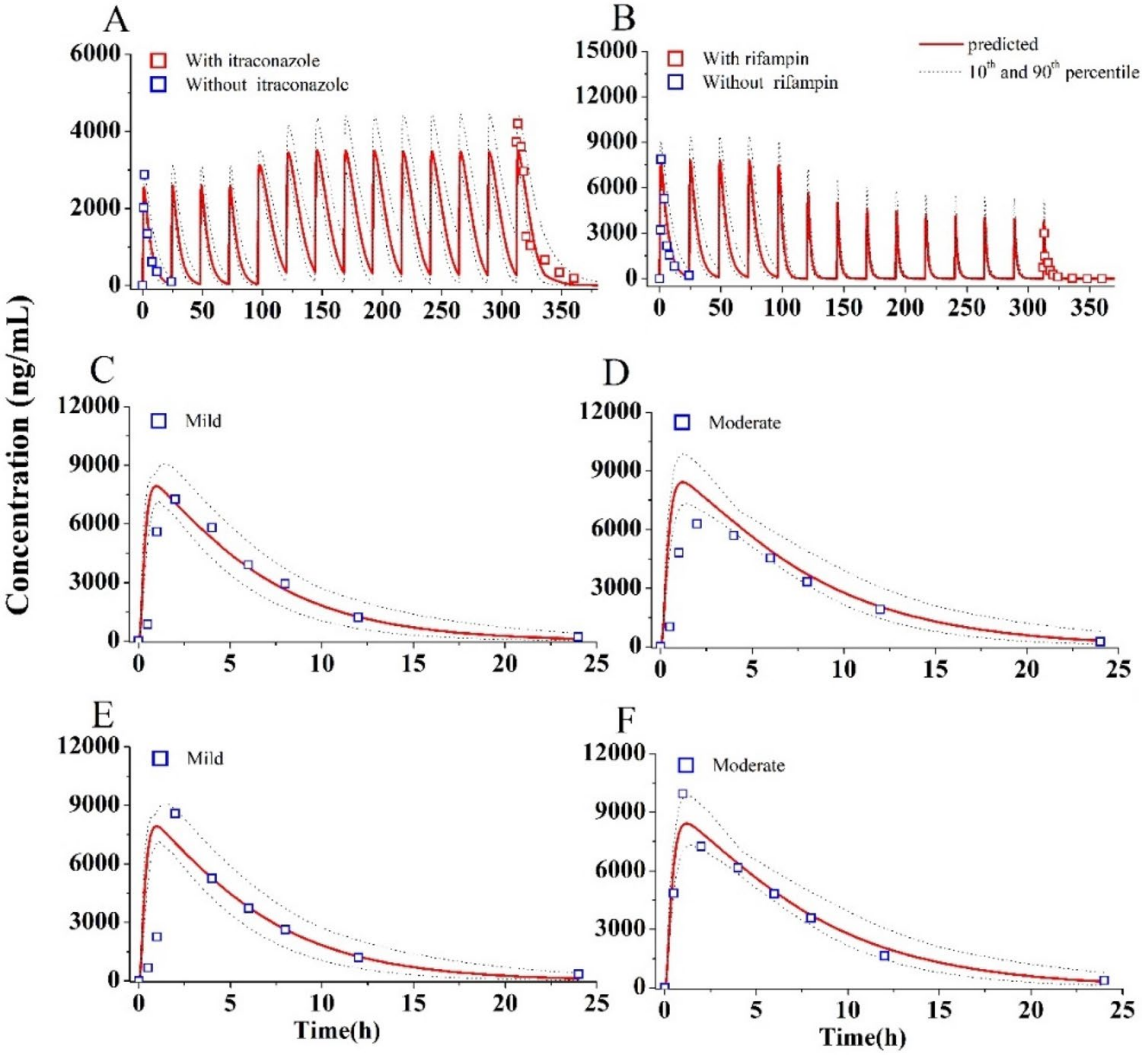
Simulations of pharmacokinetics of OLA (100 mg OD) with itraconazole (**A**), OLA (300 mg OD) with rifampin (**B**), in patients with mild (**C**) and moderate (**D**) hepatic impairment, in patients with mild (**E**) and moderate (**F**) renal impairment.

The predicted and clinically observed PK profiles and data of five CYP3A4 inhibitors have been given in supplementary Figure S1 and Table S6. The predicted PK profiles of OLA with itraconazole and rifampin are shown in Fig. 4A,B. The 90% prediction interval almost covered the variability of the clinically observed PK data. The PK ratios predicted by the PBPK model are summarized in Table 5. The predicted C_max_, C_trough_, and AUC_312-336_ ratios of OLA when dosed with itraconazole were 1.37-, 7.05- and 2.85-fold higher, respectively, compared to OLA alone. Conversely, Co-administration of OLA with rifampin resulted in a significantly predicted reduction in OLA C_max_, C_trough_, and AUC_312–336_ ratios of 0.50, 0.13 and 0.17. The predicted C_max_, C_trough_ and AUC_288–312_ ratios of OLA by the PBPK model were very close to the clinically observed values (Table 5)^3^.

**Table 5:** PK changes (geometric mean, range) of OLA under DDIs.

Parameters	OLA only (100 mg OD)	OLA + Itraconazole (100 mg OD + 200 mg OD)	Predicted^a^ ratio	Observed ratio
C_max_ (ng·mL^−1^)	2560	3513 (2998–4463)	1.37	1.41
C_trough_ (ng·mL^−1^)	39.1	275.6 (61.7–1450)	7.05	6.56
AUC_312–336_ (ng·h·mL^−1^)	14,382	40,931(28,155–67,045)	2.85	2.71
Parameters	OLA only (300 mg OD)	OLA + Rifampin (300 mg OD + 600 mg OD)	Predicted ratio	Observed ratio
C_max_ (ng·mL^−1^)	7818	3878 (2638–5222)	0.50	0.82
C_trough_ (ng·mL^−1^)	115	25(15–324)	0.13	0.05
AUC_312–336_ (ng·h·mL^−1^)	52,485	9012 (6268–13,979)	0.17	0.12

^a^Calculated by ratio of concomitant to only use.

### Simulations in patients with hepatic/renal impairment

Figure 4C–F and supplementary Table S7 show the predicated and observed PK profiles and data in patients with hepatic/renal impairment. All ratios between prediction and observation fell in the range of 0.5–2.0 (Table S7). Table 6 summarizes the predicted and observed ratios of C_max_, C_24_ and AUC_0-96_ in patients with mild, moderate and severe hepatic/renal impairment. The simulations showed that the predicted PK ratios were in agreement with the observed values^14,30^. As seen in Table 6, the geometric mean C_max_ and AUC_0-96_ were slightly increased for mild, moderate and severe hepatic/renal impairment patients within 2.0-fold. However, meaningfully increase occured for C_24_ ranging from 1.1- to 6.3-fold in all hepatic/renal impairment patients. Overall, the PBPK model could reasonably predict the influence of hepatic/renal impairment on the PK change of OLA. This indicates the developed PBPK model can predict C_max_ and C_trough_ in patients with liver/kidney impairment.

**Table 6:** PK parameter (geometric mean) ratios of OLA in patients with hepatic/renal impairment.

Parameters	Predicted ^a^			Observed ^a^		
	Mild	Moderate	Severe	Mild	Moderate	Severe
Ratio in hepatic impairment patients						
C_max_ (ng·mL^−1^)	0.99	0.87	0.94	1.14	0.74	NR
C_24_ (ng·mL^−1^)^b^	1.1	3.1	5.7	1.29	2.02	
AUC_0-96_ (ng·h·mL^−1^)	1.05	1.23	1.46	1.15	1.08	
Ratio in renal impairment patients						
C_max_ (ng·mL^−1^)	1.13	1.18	1.08	1.26	1.39	NR
C_24_ (ng·mL^−1^)^b^	1.4	3.6	6.3	1.80	2.79	
AUC_0-96_ (ng·h·mL^−1^)	1.19	1.57	1.76	1.62	1.75	

^a^Calculated by dividing normal data (300 mg OD) with mild, moderate and severe, respectively.

^b^Plasma concentration at 24 h.

### Dosage adjustment recommendation based on the PBPK model

**Figure 5:**
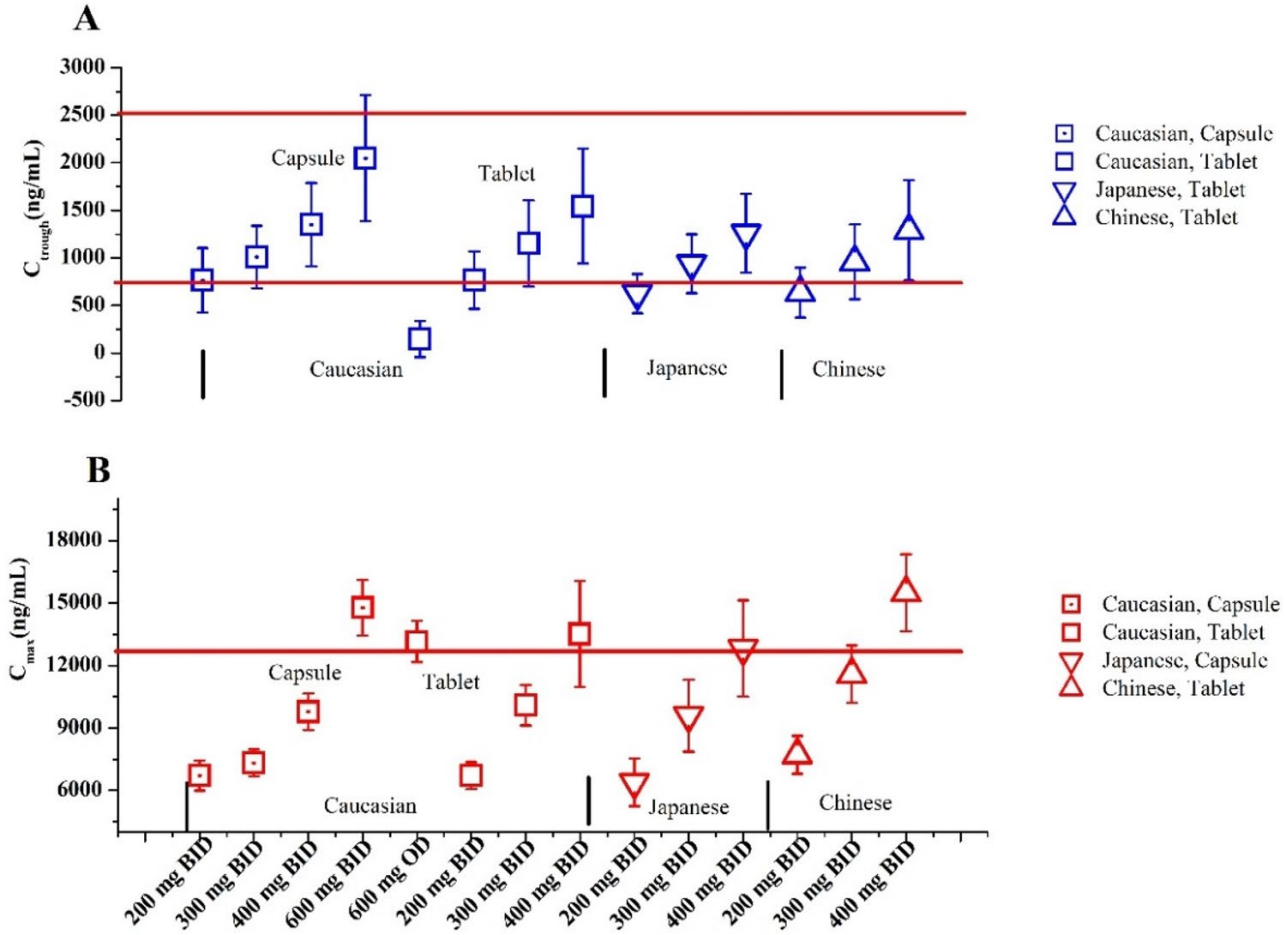
The PBPK simulations of predicted C_trough_ (**A**) and C_max_ (**B**) in Caucasian, Japanese and Chinese. Data were shown as geometric mean values and 95%CI. In the simulations, capsule and tablet were administered to Caucasian. Japanese and Chinese only took OLA tablet.

As shown in Fig. 5, the optimal dosing regimens of OLA for each of the ethnic groups were studied based on the geometric mean and 95% confidence interval (95% CI) of the predicted C_max_ and C_trough_. The PBPK model supported the optimal dosing regimens of 300 and 400 mg BID in capsule formulation, and of 300 mg BID in tablet formulation (see Fig. 5). The conclusion is in good agreement with the clinical recommendations^2,4^.

**Figure 6:**
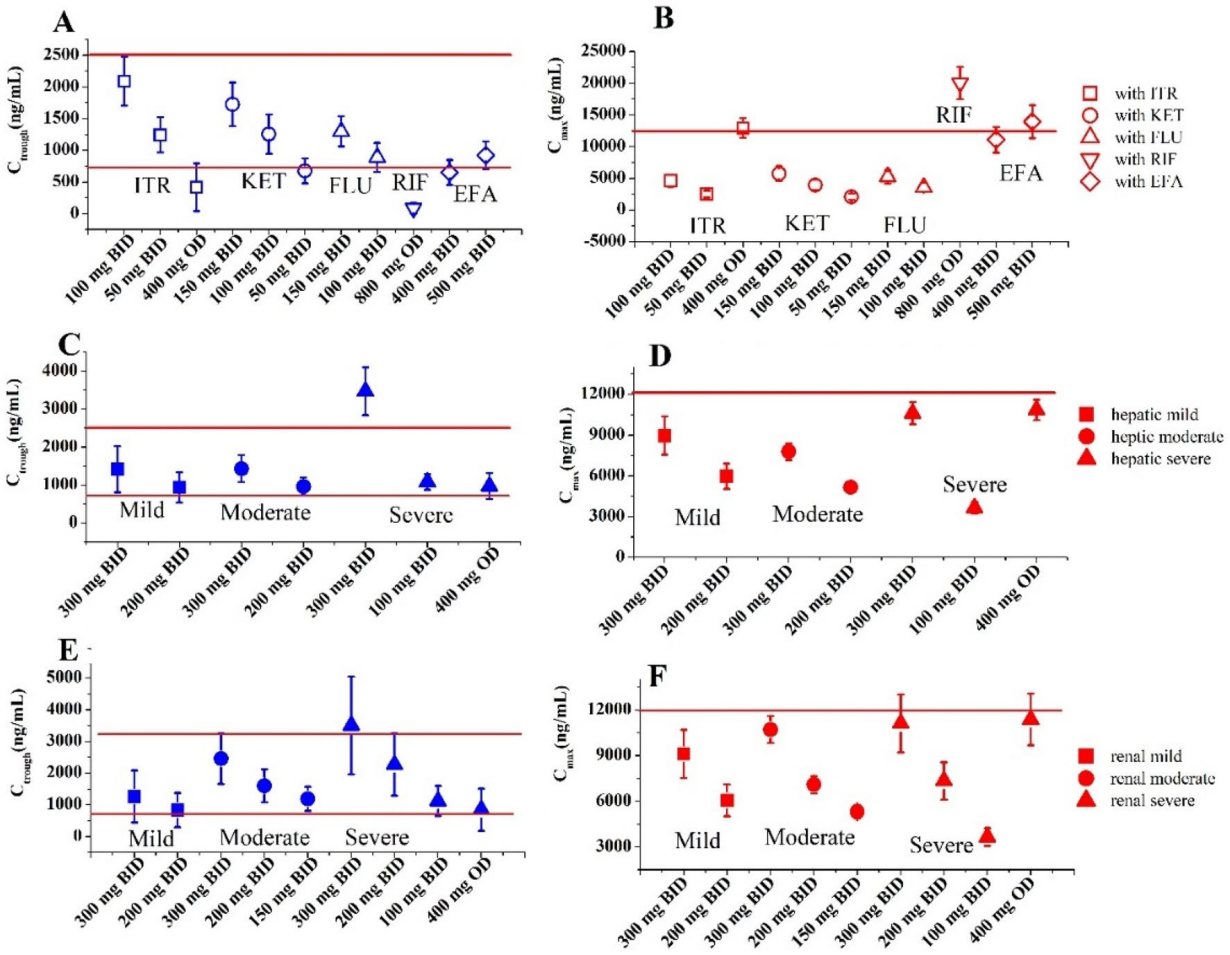
The PBPK simulations of predicted C_max_ and C_trough_ when dosed with other CYP3A4 modulators (**A**, **B**), in patients with hepatic impairment (**C**, **D**), and with renal impairment (E, F). Data were shown as geometric mean values and 95%CI. ITR: itraconazole, KET: ketoconazole, FLU: fluconazole, RIF: rifampicin, EFA: efavirenz.

Figure 6A,B shows the suitable dosing regimens for OLA under DDIs. Table 7 summarizes dosing adjustment recommendations under DDIs based on the PBPK model. As shown in Fig. 6A,B and Table 6, the PBPK model supported dose reduction when co-administered with CYP3A4 inhibitors. Dosage regimens were suggested to be adjusted to 50, 100 and 150 mg BID, respectively, when dosed with 200 mg OD itraconazole, 400 mg OD ketoconazole, and 200 mg OD fluconazole. Conversely, the PBPK model suggested to avoid concomitant use with CYP3A4 inducers (rifampicin and efavirenz). The simulations showed that dosing adjustment recommendations by the PBPK model were different from the based-AUC ratio recommendations.

**Table 7:** OLA dosing adjustment recommendations based on the PBPK model.

Scenarios	C_max_ (ng/mL, 95%CI)	C_trough_ (ng/mL, 95%CI)	AUC ratio	Based-AUC ratio recommendation	Based-model recommendation
DDI (OLA + Itraconazole 200 mg OD)					
100 mg BID	4611 (3970–5874)	2089 (1785–2754)	2.85	Reduce dose to 100 mg BID	Support dose reduction to 50 mg BID
50 mg BID	2498 (2137–3227)	1244 (1068–1221)			
400 mg OD	12,903 (11,721–14,850)	419 (314–1666)			
DDI (OLA + Ketoconazole 400 mg OD)					
150 mg BID	5737 (4939–7253)	1726 (1401–2693)	1.73	Reduce dose to 150 mg BID	Support dose reduction to 100 mg BID
100 mg BID	3956 (3388–5067)	1257 (1027–1848)			
50 mg BID	2080 (1768–2711)	675 (558–952)			
DDI (OLA + Fluconazole 200 mg OD)					
150 mg BID	5234 (4483–6616)	1299 (1030–1901)	1.4	No need to adjust dose	Support dose reduction to 150 mg BID
100 mg BID	3588 (3063–4552)	889 (751–1207)			
DDI (OLA + Rifampicin 600 mg OD)					
800 mg BID	20,024 (17,999–23,116)	86 (30–230)	0.17	Avoid concomitant use	Avoid concomitant use
DDI (OLA + Efavirenz 600 mg OD)					
400 mg BID	11,077 (9704–13,761)	650 (421–1213)	0.42	Increase dose to 600 mg BID	Not recommended
500 mg BID	13,925 (12,174–17,366)	923 (598–1432)			
Hepatic impairment					
Mild	C_max_ (ng/mL, 95%CI)	C_trough_ (ng/mL, 95%CI)	AUC ratio	Based-AUC ratio recommendation	Based-model recommendation
300 mg BID	8963 (7748–10,560)	1419 (997–2207)	1.05	No need to adjust dose	Supported dose unchanged
200 mg BID	5968 (5164–7023)	937 (661–1450)			
Moderate					
300 mg BID	7782 (7199–8457)	1430 (1148–1854)	1.23	No need to adjust dose	Support dose reduction to200 mg BID
200 mg BID	5161(4786–5616)	967 (752–1215)			
Severe					
300 mg BID	10,616 (9853–11,495)	3468 (2938–4191)	1.46	No need to adjust dose	Support dose reduction to 100 mg BID or 400 mg OD
100 mg BID	3639 (3259–4109)	1081 (914–1323)			
400 mg OD	10,853 (10,145–11,654)	976 (726–1413)			
Renal impairment					
Mild					
300 mg BID	9108 (7700–10,886)	1263 (715–2359)	1.19	No need to adjust dose	Supported dose unchanged
200 mg BID	6063 (5133–7237)	834 (475–1553)			
Moderate					
300 mg BID	10,707 (9889–11,649)	2456 (1864–3459)	1.57	Reduce dose to 200 mg BID	Support dose reduction to 150 mg BID
200 mg BID	7095 (6564–7706)	1601 (1217–2248)			
150 mg BID	5313 (4919–5765)	1192 (607–1670)			
Severe					
300 mg BID	11,114 (9493–13,288)	3506 (2477–5556)	1.76	Reduce dose to 200 or 150 mg BID	Support dose reduction to 100 mg BID
200 mg BID	7334 (6287–8731)	2274 (1616–3584)			
100 mg BID	3652 (3144–4326)	1120 (802–1751)			
400 mg OD	11,374 (9914–13,290)	847 (534–1857)			

Figure 6C–F depicts the OLA suitable dosing regimens for patients with hepatic/renal impairment. Based on the PBPK model, Table 7 summarizes dosing adjustment recommendations in patients with hepatic/renal impairment. As shown in Fig. 6C–F and Table 7, the PBPK model supported dose unchanged in patients with mild hepatic/renal impairment, and supported dose reduction in patients with moderate and severe hepatic/renal impairment.

The PBPK model suggested that dosing regimens should be reduced to 200 mg BID and 100 mg BID in patients with moderate and severe hepatic impairment, respectively. In addition, dosing regimens in patients with moderate and severe renal impairment should be reduced to 150 mg BID and 100 mg BID, respectively. However, the dosage recommendation by the PBPK model is different from the recommended dosing for patients with moderate and severe hepatic/renal impairment based on AUC ratio.

## Discussion

In this work, the PK thresholds of OLA C_max_ (< 12,500 ng/mL) and C_trough_ (772–2500 ng/mL) at steady state for clinical efficacy and safety were defined. The PBPK model of OLA can accurately predict the C_max_ and C_trough_ for two formulations in patients administered alone. This has been evidenced by multiple clinical PK study data (see Fig. 2 and Table 4). In different inter-ethnic groups, known inter-ethnic physiological differences in height and weight distribution, liver volume, CYP enzyme expression, gastrointestinal transit time, and plasma protein composition have been reported^17,33^. In this simulation, based on the paper^18^, only different liver volume and CYP3A4 abundance data between inter-ethnic populations were incorporated into the PBPK model based on literature data. The default values of the PK-Sim were used for physiological differences in height and weight distribution. The simulations found that, although the geometric mean C_max_ and C_trough_ of OLA in Chinese are lower than those in Caucasian, the predicted C_max_ and C_trough_ values are not statistically significantly different between them (Table 4).

When co-administered with itraconazole and rifampicin, there was good agreement between the PBPK prediction and clinical observation in PK profiles and data (see Table 5 and Fig. 4A,B).It is noteworthy that OLA inhibits its own metabolism through reversible time-dependent inhibition against CYP3A4 as well as enhances its own metabolism through induction of in vivo CYP3A4 expression^7^. The sensitive analysis also demonstrates that the inhibition and induction parameters of OLA had an effect on C_max_ and C_trough_ (see Fig. 3). The PBPK model incorporated CYP3A4 auto-inhibition (K_i_) and induction parameters (E_max_ and EC_50_) to ensure the predictive performance of the model. However, this may not robust for model. Recent research^34,35^ has also employed the PBPK approach to anticipate clinical DDIs involving combined CYP3A4 inhibitors and inducers, thereby providing additional validation for our PBPK approach in assessing the impact of such mixed inhibition and induction. The induction parameter of rifampicin exhibited significant inter-individual variability across various literature studies^7,36,37^.To mitigate this variability, we incorporated the induction parameter values from the most recent publication^7^ into the current model, where inhibition and induction activity of OLA were also determined concurrently. Use of rifampicin induction parameters may be reasonable based on indirect evidence that our model accurately estimated the induction of intestinal CYP3A4 by rifampicin, with predicted changes showing close agreement with experimentally determined values in humans^38^ (3.8-fold increase versus 4.4-fold change). The predicted expression of intestinal CYP3A4 after induction by rifampicin was close to the experimentally determined value in humans^38^ (3.8-fold increase vs. 4.4-fold change).

The PBPK model reasonably predicted hepatic/renal-impairment effects on the PK change of OLA (see Table 6 and Fig. 4C–F). The PBPK model slightly overpredicted PK data in patients with moderate hepatic impairment, especially on C_24_ by 1.5-fold. Prior research has demonstrated that many PBPK models tend to overpredict PK in patients with hepatic impairment^28^. This is likely because current PBPK models are unable to account for variations in absorption (such as a reduction in bile salt concentration) caused by hepatic impairment. Whereas, given the slight overestimation, fraction absorbed value was not adjusted in this study. Besides, A larger number of studies have described alterations in plasma protein concentration in patients with hepatic/renal impairment^28,30,39^, Various PPSF values (see Table S2,S3) were utilized in this study to model changes in plasma protein levels among affected patients with different types of impairment.

One published paper has reported the development of a PBPK model for OLA and its dosing considerations in DDIs using classical AUC ratios^16^. However, our study differs from this publication in two important aspects. First, we propose PK thresholds for OLA C_max_ (< 12,500 ng/mL) and C_trough_ (772–2500 ng/mL) as key parameters for clinical efficacy and safety. These thresholds, combined with the PBPK model, serve as a crucial strategy for adjusting dosing regimens in DDIs or patients with hepatic/renal impairment. Compared to relying solely on AUC ratios, this combination strategy provides better dosing adjustment, particularly for OLA, which exhibits a nonlinear dose-AUC relationship. Second, we have collected a larger dataset of clinical PK data to develop and validate our PBPK model. This model can predict OLA concentrations not only in Caucasian populations but also in Japanese and Chinese populations. To our knowledge, this is the first investigation to simulate the inter-ethnic PK of OLA across these three different populations.

For most therapeutic drug, if the exposure ratio increases or reduces by more than twofold, it may be necessary to consider clinical dosage adjustments (widely acceptable clinically significant criteria^40^). However, the systemic exposure (AUC) of OLA does not increase in direct proportion to dose within the 100–400 mg dose range^4^. The adjustment of the dosage regimen for OLA cannot be straightforward on the basis of an AUC change. The exposure–response relationship for both efficacy and safety has defined the PK thresholds of OLA C_max_ (< 12,500 ng/mL) and C_trough_ (772–2500 ng/mL). This study proposes that using PK thresholds for OLA may be a superior approach to dosing adjustment in multiple clinical scenarios based on PK thresholds of OLA C_max_ and C_trough_.

The optimal dosing regimens of OLA for each of the ethnic populations were first studied based on the geometric mean and 95%CI of predicted C_max_ and C_trough_. This strategy for optimal dosing has been proposed by Johnson et al.^41^ and Adiwidjaja et al.^18^. Based on this strategy, it was suggested that a dosing regimen consisting of 300 or 400 mg BID in capsule formulation, as well as 300 mg BID in tablet formulation, may be considered an optimal option for three different inter-ethnic patient populations (see Fig. 5). When dosed in combination with CYP3A4 inhibitors (strong and moderate), our PBPK model indicated that dosing regimens for OLA may be modified to either 50 mg BID or 150 mg BID (see Table 7 and Fig. 6A,B). Given the available approved tablets with strengths of 100 and 150 mg, our analysis suggests that dosing regimens for OLA should be adjusted to 100 mg BID when administered along with a potent CYP3A4 inhibitor and to 150 mg BID with a moderate CYP3A4 inhibitor. The recommendation is in good agreement with the clinical recommendations^2^. Conversely, when dosed in combination with CYP3A4 inducers (strong and moderate), it is not suggested to co-administer with CYP3A4 inducers based on the PBPK model (see Table 7 and Fig. 6A,B). The recommendation is also in good agreement with the clinical recommendations^2^. However, except for co-administration with rifampicin, dosing adjustment recommendations of OLA in DDIs by the PBPK model are different from the recommendations by the AUC ratios, particularly in co-administration with efavirenz. The dosing adjustment recommendations are the same for patients with mild hepatic/renal impairment between the PBPK model and AUC ratio. The dosage adjustment by the PBPK model suggested 200 mg BID and 100 mg BID (or 400 mg OD) represent an appropriate dosing schedule for patients with moderate or severe hepatic impairment, respectively. This is completely different from the recommendations based on the AUC ratio (see Table 7). This is most likely due to a more sensitive change in C_max_ and C_trough_ than in AUC. The dosage adjustment by the PBPK model suggested 150 mg BID and 100 mg BID represent a suitable dosing regimen for patients with moderate and severe renal impairment, respectively, which is lower dosing than the recommendations by AUC ratio (see Table 7). The recommendation of a dose reduction to 150 mg BID for patients with moderate renal impairment is slightly different from the clinical study (dosage reduction to 200 mg BID)^2^. The simulations in the Japanese and Chinese populations could not be validated using the available clinically observed PK data for DDIs and patients with hepatic/renal impairment, which were solely obtained from the Caucasian population. Moreover, significant differences were not found in the predicted in predicted C_max_ and C_trough_ values at steady state (Fig. 5). Therefore, the simulations for DDIs and patients with hepatic/renal impairment were exclusively conducted using the Caucasian population in this study.

However, there are certain limitations to the current model. First, the dosing adjustment for hepatically impaired patients has not been verified using any clinical study data. Another challenge associated with this PBPK approach is the absence of consideration in the PBPK model role of other inter-ethnic physiological differences, except for differences in height and weight distribution, liver volume, and CYP enzyme expression.

## Conclusion

In conclusion, the PBPK models adequately predicted the clinical PK data of OLA in three inter-ethnic populations, adequately provided DDI prediction with CYP3A4 modulators, and accurately simulated PK change in patients with hepatic/renal impairment. Finally, a dosage adjustment strategy was proposed to modify the OLA dosing regimens using this PBPK model in multiple clinical scenarios.

### Supplementary Information

Supplementary Information.

## Data Availability

All the data generated during the research is either reported in the manuscript or is provided in the supplementary file.
