# Supplementary material for: Physiologically-based pharmacokinetic modeling for optimal dosage prediction of olaparib when co-administered with CYP3A4 modulators and in patients with hepatic/renal impairment
Source: Sci Rep. 2023 Sep 25;13:16027. doi: 10.1038/s41598-023-43258-9 (PMC10519932; doi:10.1038/s41598-023-43258-9)
Supplement: Supplementary file 1 — Supplementary Information. [file 41598_2023_43258_MOESM1_ESM.pdf]

Supporting information

Physiologically-Based Pharmacokinetic Modeling for Optimal Dosage Prediction of Olaparib When Co-administered with CYP3A4 Modulators and in Patients with Hepatic/Renal Impairment

Dongmei Gao [1], Guopeng Wang [2], Honghai Wu [3], Jiawei Ren [4]✉

[1]Department of Medical Oncology, Bethune International Peace Hospital, Shijiazhuang 050082, China

[2]Zhongcai Health (Beijing) Biological Technology Development Co., Ltd., Beijing 101500, China

[3]Department of Clinical pharmacy, Bethune International Peace Hospital, Shijiazhuang 050082, China

[4] North China Electric Power University, Beijing102206, China

✉Correspondence to:

Jiawei Ren, North China Electric Power University, No.2, Beinong Road, Huilongguan, Changping District, Beijing102206, China. Tel: +86 130019810; E-mail address: rjw@ncepu.edu.cn.

Supplementary Table S1 Inputting parameters used for the PBPK models of modulators in DDI simulations

Property	Values				
	Ketoconazole[a]	Itraconazole/ Hydroxy-itraconazole[b]	Rifampicin[a]	Efavirenz[a]	Fluconazole
MW($g \cdot mol^{-1}$)	531.4	705.6/721.7	822.94	315.68	306.3
pKa	6.51(base)	3.7/2.53,4.91(base)	1.7(acid), 7.9(base)	10.1(acid)	2.03(neutral)
Log P	2.67(@pH7.4)	4.2/3.5	2.3	3.44	0.83
Solubility(μg/mL)	6.93(@pH6.5)	5.4(@pH1.2)/-	2.8(@pH7.5)	39.9	6900
P_{eff} ($\times 10^{-5}$ $cm \cdot s^{-1}$)	1.24	-	1.24	2.97	21.3
P_{app} ($\times 10^{-6}$ $cm \cdot s^{-1}$)	-	57.1/-	-	-	-
f_{up}	0.015	0.016/0.021	0.17	0.0059	0.11
Rbp	0.59	0.58/0.58	0.89	0.63	0.85
CYP3A4 CL_{int} (μl/min/pmol)	-	-	-	-	

UGT2B7 CL_{int}（L/min）	-	-	-	-	0.008
CYP3A4 V_{max} (pmol/min/pmol)	8.0	0.65/0.05	-	0.16	
CYP3A4 K_m (μM)	15.0	0.039/0.027	-	23.5	
CYP3A5 V_{max} (pmol/min/pmol)	-	-	-	0.6	
CYP3A5 K_m (μM)	-	-	-	19.1	
CYP1A2 V_{max} (pmol/min/pmol)	-	-	-	0.60	
CYP1A2 K_m (μM)	-	-	-	8.3	
CYP2A6 V_{max} (pmol/min/pmol)	-	-	-	1.0	
CYP2A6K_m (μM)	-	-	-	7.7	
CYP2B6 V_{max} (pmol/min/pmol)	-	-	-	3.5	
CYP2B6K_m (μM)	-	-	-	6.4	
CYP2C8 CL_{int} (μl/min/pmol）	-	-	-	-	
CYP2C9 CL_{int}(μl/min/pmol）	-	-	-	-	
UGT1A1 V_{max} (pmol/min/pmol)	9.37	-	-		
UGT1A1 K_m (μM)	22.3	-	-	-	
AADAC V_{max} (μM/min)	-	-	9.87	-	
AADAC K_m (μM)	-	-	195.10	-	
P-gp V_{max} (μM/min)	-	-	0.036	-	
P-gp K_m (μM)	-	-	55.0	-	
OATP1B1 V_{max} (μM/min)	-	-	0.086	-	
OATP1B1K_m (μM)	-	-	1.5	-	
CL_R(L/h)	GFR*f_u				
GFR fraction	1.0	1.0	1.0	1.0	0.2
K_p scale	-	0.6/2.0	5.0	-	-
Partition coefficients	Rodgers and Rowland				
Cellular permeabilities	PK-Sim Standard				
Weibull time (min)	120				
Weibull shape	0.92				

[a]: The modeling parameters were built in the OSP library of PK-Sim.
[b]: The modeling parameters were taken from the reference S1.

The clinical observed PK data were taken from the references S2 (ketoconazole), S1(itraconazole), S3(rifampicin), S4 (efavirenz), and S5 (fluconazole).

Supplementary Table S2 Physiological parameters used in patients with hepatic organ impairment in the PBPK model

Physiological parameters	patients with liver impairment			Source
	Mild	Moderate	Severe	
Hematocrit Value	0.39	0.37	0.35	
Renal blood flow (mL/min)	254.0	187.6	138.5	[S6]
GFR (mL/min)	123.2	86.3	44.4	
Hepatic arterial (mL/min)	31.1	35.5	42.2	
Liver volume (L)	2.14	1.69	1.45	
Gastric residence time (h)	0.30	0.35	0.38	[S7]
Colon transit time (h)	88.4	88.4	88.4	
CYP3A4 abundance in the liver (pmol/mg)	108	56	31	
CYP3A4 activity for CYP3A4 $CL_{int,u}$	0.044	0.037	0.018	[S6, S7]
Albumin (g/L)	39.0	27.0	24.6	[S7,S8]
PPSF	1.05	1.44	1.55	Calculated

Supplementary Table S3 Physiological parameters used in patients with renal organ impairment in the PBPK model

Physiological parameters	Physiological parameters disease patients by stage			Source
	Mild	Moderate	Severe	
GFR（mL/min）	60-89	30-60	<30	[S9]
Hematocrit Value	0.45	0.42	0.38	
Albumin (g/L)	41.0	38.1	34.9	[S7]
Gastric emptying time (h)	0.25	0.25	0.40	
CYP3A4 abundance (pmol/mg protein)	137.0	95.2	87.3	
Hepatic arterial blood flow(mL/min/100 g organ)	18.0	18.0	6.66	[S6]
P-gp activity(μL/min/million cells^{-1})	0.48	0.32	0.13	
Renal blood flow (mL/min/100 g organ)	172.0	115.1	72.9	Calculated based on literature[S10]
Kidney volume (L)	0.39	0.32	0.23	
Small intestinal transit time (h)	2.1	2.1	2.9	[S10]
CYP3A4 activity(μL/min/pmol)	0.044	0.031	0.028	Calculated based on literature[S7]
PPSF	1.0	1.07	1.15	Calculated

Supplementary Table S4 Characteristics comparisons of patients with hepatic impairment in virtual and clinical populations

	Mild		Moderate		Severe	
	clinical	virtual	clinical	virtual	clinical	virtual
Number of subjects	10	10	8	10	-	10

Age (year, range)	58 (41-70)	60 (41-70)	67 (52-78)	69 (52-78)	-	66(52-78)
Proportion of female (%)	40	40	25	30	-	50
Race	Caucasian	Caucasian	Caucasina:7 ; Asian:1	Caucasian	-	Caucasian
ALT(μkat/L, range)	1.3 (0.1-4.4)	*	0.7 (0.3-2.3)	*	-	*
AST(μkat/L, range)	1.0 (0.4-2.3)	*	1.0 (0.6-3.2)	*	-	*
Bilirubin(μmol/L, range)	12.8 (3-15)	*	24.5 (10-45)	*	-	*
Mean albumin (g/L, range)	39.0 (30-46)	*	27.0 (2-37)	*	-	*

-: not reported data; *: cannot be set values in PK-Sim.

Supplementary Table S5 Characteristics comparisons of patients with renal impairment in virtual and clinical populations

	Mild		Moderate		Severe	
	clinical	virtual	clinical	virtual	clinical	virtual
Number of subjects	15	15	14	14	-	10
Age (year, range)	63 (50-75)	60 (50-75)	68 (32-76)	63 (32-76)	-	63 (32-76)
Proportion of female (%)	53	53	50	50	-	50
Race	Caucasina:14 ; Asian:1	Caucasian	Caucasian	Caucasian	-	Caucasian
GFR（mL/min, SD）	60.1 (8.1)	59 (13)	41.7 (5.8)	39 (13)	-	14 (4)

-: not reported data.

Supplementary Table S6 The mean observed and predicted PK data for the modulators according to their respective PBPK model

Drug	Parameters	Predicted	Observed	Predicted/Observed
Ketoconazole	C_{max} (μg·mL^{-1})	7289.1	6240.0	1.17
	AUC_{0-48} (μg·h·mL^{-1})	5235.9	4687.2	1.12
	T_{max} (h)	1.7	1.5	1.13
Itraconazole	C_{max} (ng·mL^{-1})	765.6	556.0	1.38
	AUC_{0-48} (ng·h·mL^{-1})	5511.3	4500.5	1.22
	T_{max} (h)	1.9	2.0	0.95
Hydroxy-Itraconazole	C_{max} (ng·mL^{-1})	723.4	639.0	1.13
	AUC_{0-48} (ng·h·mL^{-1})	11676.6	13128.9	0.89
	T_{max} (h)	3.5	4.0	0.88
Rifampicin	C_{max} (ng·mL^{-1})	10977.2	9540.0	1.15
	AUC_{0-24} (ng·h·mL^{-1})	63191.4	62861.3	1.01
	T_{max} (h)	1.2	1.5	0.80
Efavirenz	C_{max} (ng·mL^{-1})	4791.4	5240.0	0.91
	$AUC_{336-360}$ (ng·h·mL^{-1})	11284.6	11644.5	0.97
	T_{max} (h)	340	341	1.00
Fluconazole	C_{max} (ng·mL^{-1})	2820	2649	1.06
	AUC_{0-48} (μg·h·mL^{-1})	109.0	106.5	1.02
	T_{max} (h)	1.5	2.5	0.6

Supplementary Table S7 The geometric mean observed and predicted PK data in patients with hepatic/renal impairment

Hepatic impairment	Parameters	Predicted	Observed	Predicted/Observed
Normal 300 mg OD	C_{max} (ng·mL^{-1})	7692	7300	1.05
	T_{max} (h)	1.1	1.5	0.73
	AUC_{0-96} (ng·h·mL^{-1})	53090	52300	1.02
Mild 300 mg OD	C_{max} (ng·mL^{-1})	7585	8300	0.91
	T_{max} (h)	1.1	2.1	0.52
	AUC_{0-96} (ng·h·mL^{-1})	55533	60300	0.92
Moderate 300 mg OD	C_{max} (ng·mL^{-1})	6702	6400	1.05
	T_{max} (h)	1.2	1.5	0.80
	AUC_{0-96} (ng·h·mL^{-1})	65548	56000	1.17
Severe 300 mg OD	C_{max} (ng·mL^{-1})	7232	-	-
	T_{max} (h)	1.05	-	-
	AUC_{0-96} (ng·h·mL^{-1})	77501	-	-
Renal impairment	Parameters	Predicted	Observed	Predicted/Observed
Normal 300 mg OD	C_{max} (ng·mL^{-1})	7012	7200	0.97
	T_{max} (h)	0.95	2.0	0.48
	AUC_{0-96} (ng·h·mL^{-1})	45962	43700	1.05
Mild 300 mg OD	C_{max} (ng·mL^{-1})	7933	9100	0.87
	T_{max} (h)	0.95	1.6	0.59

	AUC_{0-96} ($ng \cdot h \cdot mL^{-1}$)	54630	70600	0.77
	C_{max} ($ng \cdot mL^{-1}$)	8246	10000	0.82
Moderate 300 mg OD	T_{max} (h)	1.2	2.0	0.60
	AUC_{0-96} ($ng \cdot h \cdot mL^{-1}$)	72348	76400	0.95
	C_{max} ($ng \cdot mL^{-1}$)	7565	-	-
Severe 300 mg OD	T_{max} (h)	1.5	-	-
	AUC_{0-96} ($ng \cdot h \cdot mL^{-1}$)	80876	-	-

Figure S1 The mean predicted and observed plasma concentration-time profiles of ketoconazole (A), itraconazole (B), rifampicin (C), efavirenz (D), and fluconazole (E) in healthy humans. The blue squares (□) and red up-triangles (△) refer to the clinically observed pharmacokinetic data.

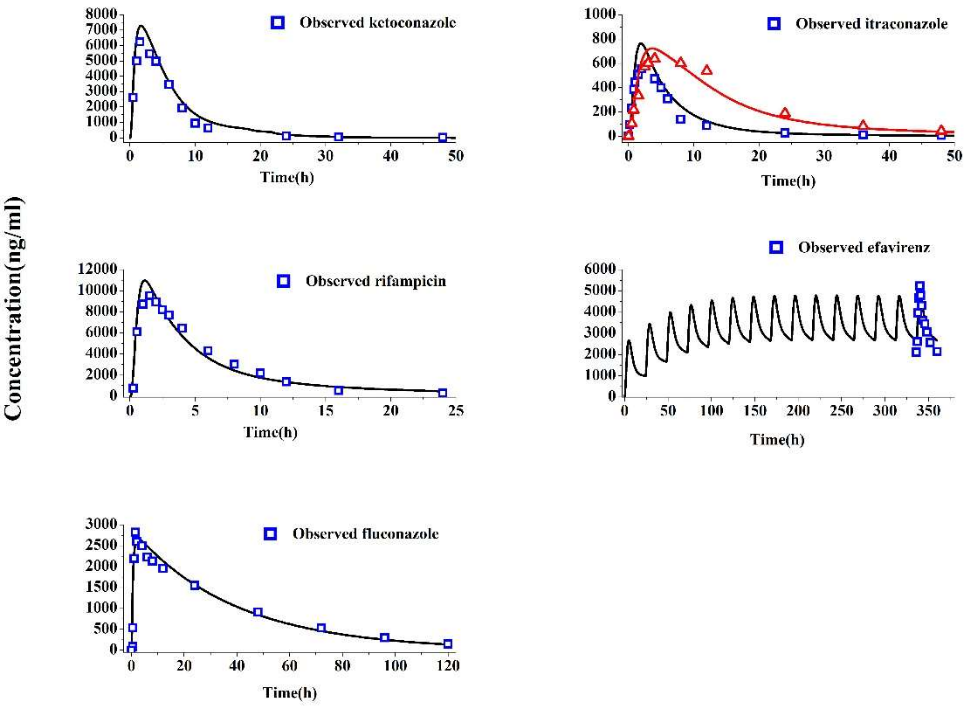

References

[S1] Chen, Y. *et al.* Development of a physiologically based pharmacokinetic model for itraconazole pharmacokinetics and drug–drug interaction prediction. *Clin Pharmacokinet* **55**, 735-749 (2016).

[S2] Daneshmend, T. K. & Warnock, D. W. Clinical pharmacokinetics of ketoconazole. *Clin Pharmacokinet* **14**, 13-34 (1988).

[S3] Hanke, N. *et al.* PBPK models for CYP3A4 and P-gp DDI prediction: a modeling network of rifampicin, itraconazole, clarithromycin, midazolam, alfentanil, and digoxin. **7**, 647-659 (2018).

[S4] Ji, P. *et al.* Pharmacokinetic interaction between efavirenz and carbamazepine after

multiple-dose administration in healthy subjects. *J Clin Pharmacol* **48**, 948-956 (2008).

[S5] Al-Mahroos, M. I. A., Al-Tamimi, D. J. J., Al-Tamimi, Z. J. J. & Ibraheem, J. J. Clinical pharmacokinetics and bioavailability study between generic and branded fluconazole capsules. *Am J Pharm Educ* **11** (2021).

[S6] Willmann, S. *et al.* Applications of physiologically based pharmacokinetic modeling of rivaroxaban—Renal and hepatic impairment and drug-drug interaction potential. J Clin Pharmacol **61**, 656-665 (2021).

[S7] Heimbach, T. *et al.* Physiologically-based pharmacokinetic modeling in renal and hepatic impairment populations: a pharmaceutical industry perspective. *Clin Pharmacol Ther* **110**, 297-310 (2021).

[S8] Rolfo, C. *et al.* Pharmacokinetics and safety of olaparib in patients with advanced solid tumours and mild or moderate hepatic impairment. *Brit J Clin Pharmaco* **86**, 1807-1818 (2020).

[S9] Rolfo, C. *et al.* Pharmacokinetics and safety of olaparib in patients with advanced solid tumours and renal impairment. *Clin Pharmacokinet* **58**, 1165-1174 (2019).

[S10] Malik, P. R. *et al.* A physiological approach to pharmacokinetics in chronic kidney disease. *J Clin Pharmacol* **60**, S52-S62 (2020).
